# Whole-exome mutational landscape and molecular marker study in mucinous and clear cell ovarian cancer cell lines 3AO and ES2

**DOI:** 10.1186/s12885-023-10791-9

**Published:** 2023-04-06

**Authors:** Jianxiong Li, Huaguo Liang, Wentao Xiao, Peng Wei, Hongmei Chen, Zexin Chen, Ruihui Yang, Huan Jiang, Yongli Zhang

**Affiliations:** 1 Longgang District Maternity & Child Healthcare Hospital of Shenzhen City (Longgang Maternity and Child Institute of Shantou University Medical College), Shenzhen, PR China; 2 School of Life Sciences and Biopharmaceutics, Guangdong Pharmaceutical University, Guangzhou, PR China

**Keywords:** Ovarian cancer, Whole-exome sequencing, CCDC170, THBS2, COL14A1, Molecular markers

## Abstract

**Background:**

Ovarian cancer is one of the most lethal cancers in women because it is often diagnosed at an advanced stage. The molecular markers investigated thus far have been unsatisfactory.

**Methods:**

We performed whole-exome sequencing on the human ovarian cancer cell lines 3AO and ES2 and the normal ovarian epithelial cell line IOSE-80. Molecular markers of ovarian cancer were screened from shared mutation genes and copy number variation genes in the 6q21-qter region.

**Results:**

We found that missense mutations were the most common mutations in the gene (93%). The MUC12, FLG and MUC16 genes were highly mutated in 3AO and ES2 cells. Copy number amplification occurred mainly in 4p16.1 and 11q14.3, and copy number deletions occurred in 4q34.3 and 18p11.21. A total of 23 hub genes were screened, of which 16 were closely related to the survival of ovarian cancer patients. The three genes CCDC170, THBS2 and COL14A1 are most significantly correlated with the survival and prognosis of ovarian cancer. In particular, the overall survival of ovarian cancer patients with high CCDC170 gene expression was significantly prolonged (P < 0.001). The expression of CCDC170 in normal tissues was significantly higher than that in ovarian cancer tissues (P < 0.05), and its expression was significantly decreased in advanced ovarian cancer. Western blotting and immunofluorescence assays also showed that the expression of CCDC170 in ovarian cancer cells was significantly lower than that in normal cells (P < 0.001, P < 0.01).

**Conclusions:**

CCDC170 is expected to become a new diagnostic molecular target and prognostic indicator for ovarian cancer patients, which can provide new ideas for the design of antitumor drugs.

**Supplementary Information:**

The online version contains supplementary material available at 10.1186/s12885-023-10791-9.

## Background

Ovarian cancer (OC) is the fifth leading cause of cancer death in women, with approximately 300,000 new cases and more than 180,000 deaths annually worldwide [1, 2]. At present, gynecological ovarian cancer has a high malignancy and younger trend [3]. Although early-stage ovarian cancer has a good prognosis with surgical treatment and chemotherapy, the early symptoms of ovarian cancer are not obvious, and the sensitivity and specificity of molecular markers used for early diagnosis are poor, resulting in more than 70% of ovarian cancer patients being diagnosed at an advanced stage [4, 5]. A high recurrence rate of advanced ovarian cancer leads to poor prognosis, with a 5-year survival rate below 45% [6, 7].

Epithelial ovarian cancer (EOC) is the most common type of ovarian cancer. According to the morphological criteria of tumor cells, it is divided into four main histological subtypes: serous ovarian carcinoma (SOC), mucinous ovarian carcinoma (MOC), ovarian endometrioid carcinoma (OEC) and ovarian clear cell carcinoma (OCCC) [8]. High tumor heterogeneity among different subtypes and within a single tumor is one of the main causes of treatment failure [9, 10]. Further research is needed to expand and strengthen this classification and characterize less common subtypes. Whole exome sequencing (WES) can reveal the mutation landscape of heterogeneous diseases and provide new methods for the pathogenesis, diagnosis and treatment of diseases [11]. For example, Kim SI et al. [12] revealed through WES studies that PIK3CA, ARID1A and KRAS are frequently mutated in ovarian clear cell carcinoma. Li C et al. [13] identified c-MYC gain as a potential target for BET inhibitors. At the same time, Kim S et al. [14] also revealed that MUC3 A is associated with the progression of ovarian cancer, and MUC3 A can be used as a potential biomarker for the diagnosis of ovarian cancer. In addition, recent studies have shown that ESR1-CCDC170 recurrent gene fusion only exists in ovarian cancer patients with short term survival [15]. High expression of THBS2 is associated with an increased risk of hematogenous and lymphatic metastasis in ovarian cancer [16]. Studies have confirmed that COL14A1 is associated with the progression and metastasis of breast cancer, but there are few reports on the occurrence and development of ovarian cancer [17]. Despite efforts to understand the pathogenic mutations and development of ovarian cancer, the pathogenesis is still insufficient.

Malignant tumors are often accompanied by copy number changes and gene mutations [18]. Studies have reported that in ovarian cancer and some other tumors, such as liver cancer, breast cancer and cervical cancer, copy number deletion or amplification on the long arm of chromosome 6 is relatively common [19–21]. Therefore, bioinformatics analysis and experimental verification of copy number changes in the pathogenicity-sensitive region of chromosome 6 in ovarian cancer will help us find more accurate molecular markers for ovarian cancer diagnosis, treatment, targeted therapy and prognosis evaluation. This study gives insights into the potential new genetic mechanism of ovarian cancer, which facilitate novel drug development.

In this study, we performed whole-exome sequencing of ovarian cancer cell lines 3AO and ES-2 and normal ovarian epithelial cell line IOSE-80. Single nucleotide variation (SNV) and copy number variation (CNV) analyses were integrated to better elucidate the genomic landscape of ovarian cancer. Meanwhile, hub genes were screened out among the shared mutated and copy number altered genes to systematically identify the molecular drivers associated with the prognosis of this disease.

## Methods

### Cell lines and reagents

Human ovarian cancer cell line ES-2 cells and human normal ovarian epithelial cell line IOSE-80 cells were purchased from Suyan Biotech (Guangzhou, China). Human ovarian cancer cell line 3AO cells were purchased from Mingjing Biology (Shanghai, China). 3AO and IOSE-80 cells were cultured in RPMI1640 medium containing 10% fetal bovine serum (FBS) (Zhejiang Tianhang Biotechnology Co.,Ltd., Zhejiang, China), and 1% penicillin/streptomycin (P/S) at 37 °C in a humidified atmosphere of 5% CO2. ES2 cells were cultured with McCOY’5 A medium (BasalMedia, Shanghai, China), and the other conditions were consistent with the above cells.

### Whole exome sequencing

DNA was extracted from 1 × 10^6^ cell pellet. The concentration of DNA samples was detected by Qubit fluorescence quantitative instrument. The integrity of DNA samples was detected by 1% agarose gel electrophoresis. Covaris instrument was used to breakup the DNA samples by ultrasonic wave, and 300–400 bp fragments were selected by magnetic beads. The DNA fragment ends were repaired, “A” was added, and the adapter was connected. The PCR reaction system was prepared, and the reaction program was set up to amplify the connected products. The amplified products were screened by magnetic beads. After the PCR product was denatured into a single strand, the cyclization reaction system was prepared, and the single-strand ring product was obtained by fully mixing the temperature-appropriate reaction for a certain time. After digesting the linear DNA molecules that were not cyclized, the final library was obtained. The verified DNA library was sequenced on the BGISEQ-500 platform (BGI-Shenzhen, China).

### Mutation calling

Trim the adapter in the FASTQ file. Poor quality readings or bases were deleted. Then, the clean data was compared with the human reference genome (hg38) using Burrows-Wheeler Aligner (BWA) [22]. Picard is used to delete duplicate sequence reads. Rearrange using the Genome Analysis Toolkit (GATK). Single nucleotide polymorphism (SNP) and insertion-deletion (Indel) are calling using GATK. Mutation effect predictor (VEP) is used for functional annotation of somatic mutations. All candidate variants were filtered against public databases including the 1000 Genomes Project, the Single Nucleotide Polymorphism Database (dbSNP), and the NHLBI exome sequencing project (ESP) 6500.

### Mutational signature analysis

There are six variant types of single base substitution, as follows: C > A/G > T, C > G/G > C, C > T/G > A, T > A/A > T, T > C/A > G and T > G/A > C. Unsupervised clustering is used to decompose and cluster 96 mutation frequencies, which are divided into different mutation features according to the way of point mutation. R package Mutational Pattern [23] was used to compare 30 known mutational features in the COMSIC database to observe the similarity.

### Analysis of somatic cell copy number variation

CNVkit software [24] was used to call the somatic cell copy number of the BAM file obtained above. The “cns” file was converted into a “seg” file, and the region with significant copy number changes was calculated using GISTIC 2.0 software. Regions with |log2ratio| greater than 0.25 are considered copy number change regions.

### Functional enrichment analysis

Gene ontology (GO) enrichment analysis and Kyoto Encyclopedia of Genes and Genomes (KEGG) enrichment analysis were performed using the annotation, visualization and integrated discovery online tool (DAVID, https://david.ncifcrf.gov/) [25]. GO analysis is a commonly used method for defining genes and their RNA or protein products to identify the unique biological characteristics of high-throughput transcriptome or genomic data. Go analysis included biological process (BP), molecular function (MF) and cellular component (CC) analysis. KEGG enrichment analysis revealed the biological pathways of mutant genes. The Bioinformatics (http://www.bioinformatics.com.cn/srplot) is a free online platform for data analysis. The bubble diagram was drawn through the Bioinformatics.

### Protein-protein interaction (PPI) network generation and module analysis

Based on genetic variation, shared mutant genes (a total of 2547 genes) and copy number variation genes (a total of 442 genes) were collected from the two cell lines. To better understand the function of mRNA for the above genes, PPI information was evaluated using the online STRING database. Enter the gene symbol in the STRING database, select the organism as “Homo sapiens”, select the minimum interaction score as “medium confidence (0.400)”, and then export the data as “TSV format”. TSV format data were integrated into gene networks associated with protein targets and visualized using Cytoscape 3.6.0. MCODE clusters a given network based on topology to find densely connected regions [26]. The MCODE application in Cytoscape screened hub genes (degree cutoff = 2, maximum depth = 100, k-core = 2, node score cutoff = 0.2).

### Differential expression and survival analysis

GEPIA (Gene Expression Profiling Interactive Analysis, http://gepia.cancer-pku.cn/) online tools provide fast and customizable functionality based on TCGA and GTEx data [27]. Differential expression analysis of hub genes was performed using the GEPIA online tool based on the ovarian cancer cohort in the TCGA database. Enter the gene symbol in the GEPIA online tool, select “Expression DIY”, q-value Cutoff is 0.01, Dataset selects “OV”.

Kaplan Meier plotter (http://kmplot.com/analysis/) enables univariate and multivariate Cox proportional hazard survival analyses using data from genomic, transcriptomic, and proteomic studies [28]. Enter a gene symbol in the Kaplan Meier plotter to perform an overall survival (OS) analysis of the hub gene using survival data including ovarian cancer patients in the TCGA and GEO databases.

### Western blotting analysis

Cells were lysed with RIPA buffer (Beyotime, China) to obtain total protein, and protein concentration was determined by BCA method. An equal amount of the sample was separated by sodium dodecyl sulfate-polyacrylamide gel electrophoresis and the protein was transferred onto a polyvinylidene fluoride (PVDF) membrane (Millipore, Burlington, USA). After blocking with 5% skim milk powder (Beyotime, China) at room temperature for 2 h, the membrane and primary antibody were incubated overnight at 4 °C. Primary antibodies: CCDC170 (coiled-coil domain-containing 170) (#bs-15255R, Bioss, Beijing, China), COL14A1 (collagen type XIV alpha 1 chain) (#AF0573, Affinity Biosciences, Cincinnati, OH, USA) and THBS2 (thrombospondin 2) (#bs-7524R, Bioss, Beijing, China). Then it was incubated with horseradish peroxidase secondary antibody (Beyotime, China) at room temperature for 2 h. Protein bands were detected by enhanced chemiluminescence.

### Cell immunofluorescence analysis

Cell slides were prepared in 24-well plates at 3 × 10^4^ cells/well. After the cell state was stable, the slide was fixed with 4% paraformaldehyde. Then 0.5% Triton X-100 was used to permeabilize the cells for 20 min at room temperature to break the membrane. After blocking with 5% BSA for 30 min at room temperature, the primary antibody was incubated in a wet box at 4 °C overnight. Primary antibodies: CCDC170, COL14A1 and THBS2. Then it was incubated with fluorescent secondary antibody in the dark for 1 h, and the nucleus was re-stained with DAPI. The plates were blocked with an anti-fluorescence quenching sealer, and the collected images were observed under a fluorescence microscope.

### Statistical analysis

All experimental results were expressed as the mean ± standard deviation (SD) of triplicate samples. The gray values of protein bands and the fluorescence intensity of immunofluorescence were analyzed using Image J software (National Institutes of Health, Bethesda, MD, USA). Statistical analysis was performed using IBM SPSS Statistics 26.0 (IBM, Armonk, USA) and GraphPad Prism 8.0 (GraphPad Software, La Jolla, CA, USA). One-way analysis of variance and Dunnett multiple comparison test were used to determine the differences between the average values of different groups. Significance was determined at values *P < 0.05, **P < 0.01, ***P < 0.001.

## Results

### Somatic mutations and characteristics of 3AO and ES2 cells

**Fig. 1:**
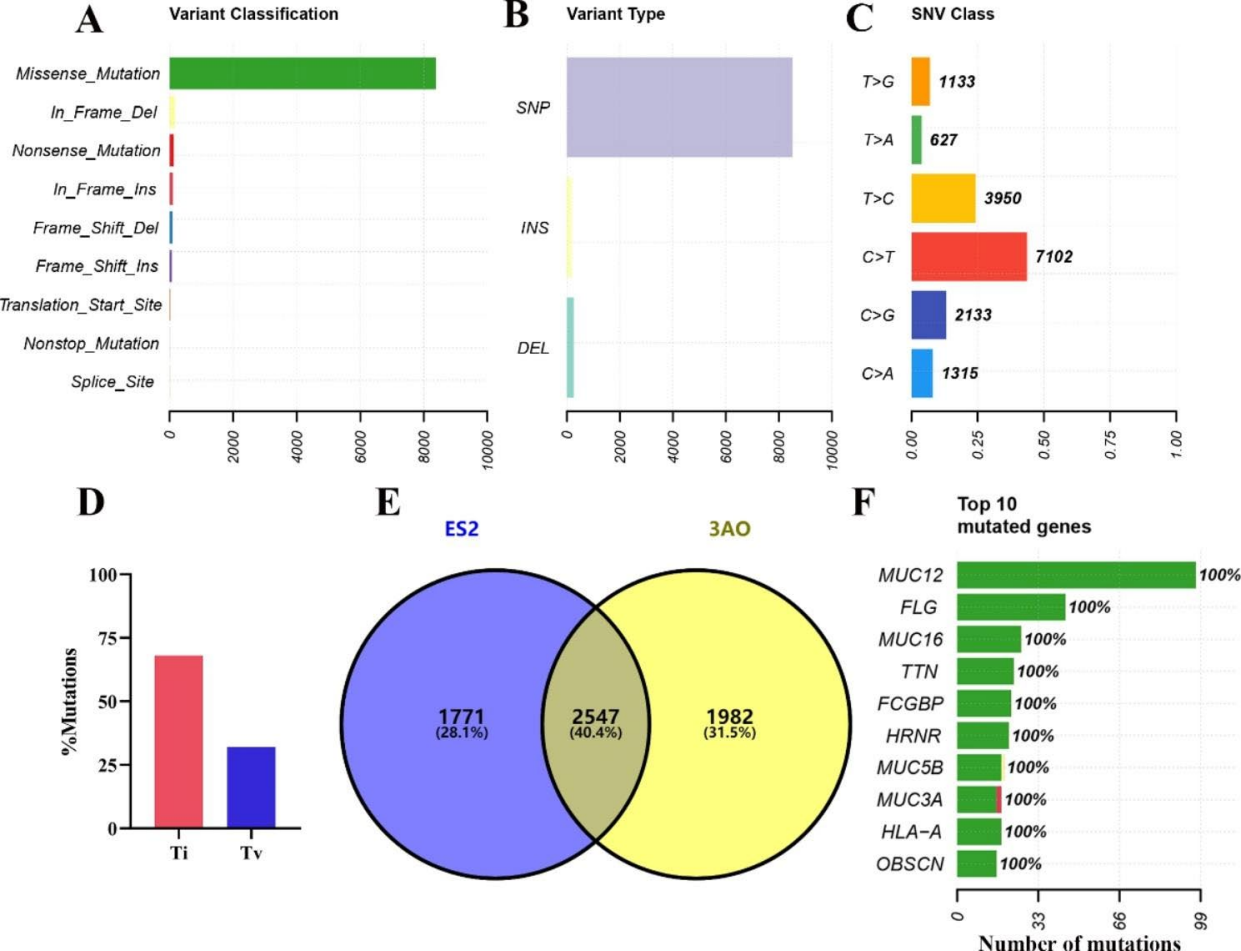
The landscape of single nucleotide variation, insertion and deletion in 3AO and ES2 cells. (**A**) Statistical graphs of different variants. (B) Statistical graph of single nucleotide variation, insertion and deletion. (**C**) The proportion and number of 6 base substitutions were shown. (**D**) The ratio of base transition and transversion. (**E**) Venn diagram of mutant genes in 3AO and ES2 cells. (**F**) Showed the top 10 most mutated genes. Green represents missense_Mutation, red represents Nonsense_Mutation, and yellow represents In_Frame_Del. The X-axis indicates the number of mutations

We performed whole-exome sequencing on normal ovarian epithelial cells IOSE-80 and ovarian cancer cell lines 3AO and ES2. The average sequencing depth reached 123.5X, sequencing base quality Q30 > 90%. A total of 8956 somatic mutations were identified in two ovarian cancer cell samples, including Missense Mutation, Frame Shift Del, Frame Shift Ins, In Frame Del, In Frame Ins, Nonsense Mutation, Nonstop Mutation, Splice Site and Translation Start Site (Fig. 1A-B).

Among them, 8688 were single nucleotide polymorphisms (SNPs) and 268 were somatic insertions and deletions. The main variation observed was missense mutations (93%) (Fig. 1A). T > C and C > T were the two most common base substitution types (Fig. 1C). The ratio of base transition to base transversion (Ti/Tv) was 2.26 (Fig. 1D). At the same time, to observe the shared mutated genes of ovarian cancer 3AO and ES2 cells, we drew a Venn diagram based on the mutation sites. In 3AO and ES2 cells, there were 6300 total mutant genes and 2547 shared mutant genes (Fig. 1E). The MUC12, FLG, MUC16, TTN and FCGBP genes were highly mutated (Fig. 1F).

As reported in the literature, TP53 mutations are not apparent in mucinous and clear cell ovarian cancer [29]. Mutations in MUC16 (carbohydrate antigen 125, CA125) have been shown to be associated with tumor burden and progression in patients [30]. MUC12, also a member of the mucin family, is overexpressed in renal cell carcinoma and promotes tumor metastasis via c-Jun/TGF-β signaling [31]. We observed mutations in the top 10 mutated genes in three ovarian cancer cohorts in the cBioPortal database. The mutation frequencies of FLG, MUC16, TTN, FCGBP and HRNR were more than 10%, and the main mutation type was copy number amplification (Additional file 1: Fig S1). The detailed analysis showed that the MUC12 gene had frequent missense mutations near sequences 250, 1700, 3200 and 4200 (Additional file 1: Fig S1).

### Mutational signature comparison of 3AO and ES2 cells

The unsupervised clustering method is used to analyze the characteristics of point mutation. Our data summarize Signature A and Signature B (Additional file 2: Fig S2). In mucinous ovarian cancer 3AO cells, the proportions of Signature A and Signature B were basically the same. Signature B was dominant in clear cell ovarian cancer ES2 cells (Additional file 2: Fig S2). The mutational signature was then compared with 30 known mutational signatures in the COMSIC database using the R package Mutational Pattern. The mutation characteristics of 3AO and ES2 cells had the highest cosine similarity with Signature.5 (Additional file 2: Fig S2). We also found a meaningful Signature.6 with relatively high cosine similarity that is worthy of attention. Signature.5 has been found in most cancers, but the cause is unclear. Signature.6 is associated with defective DNA mismatch repair and often occurs in microsatellite unstable tumors.

### Somatic cell copy number variation and the carcinogenic signaling pathway in ovarian cancer cell lines

**Fig. 2:**
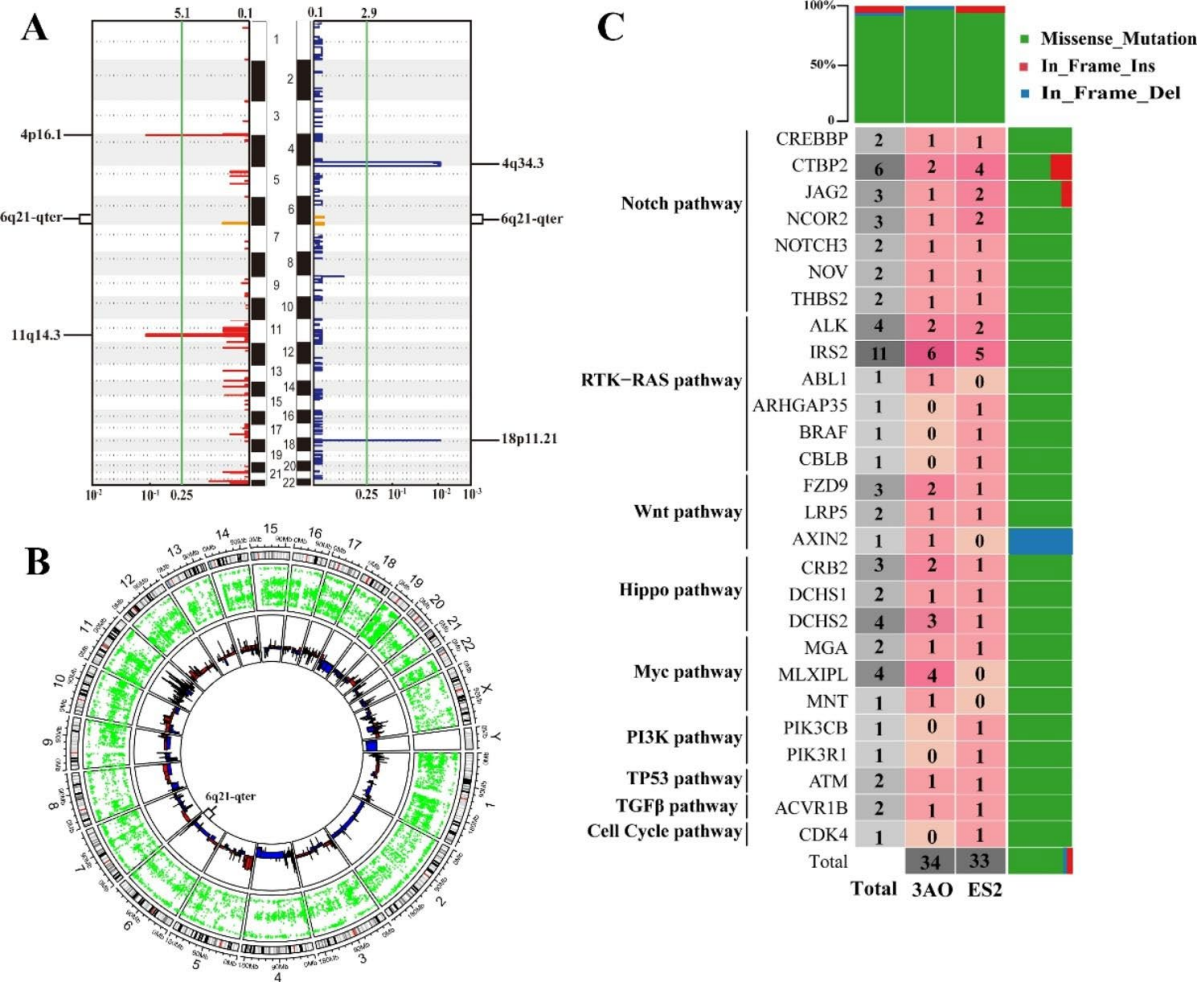
Copy number variation, oncogenic signaling pathway and gene variation in ovarian cancer cell lines. (**A**) Significant copy number variations in ovarian cancer cell lines. Red on the left represents amplification and blue on the right represents loss. Orange represents the copy number variation of the 6q21-qter region. (**B**) Ovarian cancer cell line mutation landscape circus diagram. The first circle represents the chromosome, the second circle of green dots represents single nucleotide variation, and the third circle shows copy number amplification (red) or deletion (blue). From outer ring to inner ring. (**C**) Distribution and mutation types of mutant genes in ten carcinogenic signaling pathways

Repeated somatic copy number amplification at 4p16.1, 11q14.3 and 6q21-qter and loss of copy number at 4q34.3 and 18p11.21 occurred in ovarian cancer cells (Fig. 2A, Additional file 3: Fig S3). Copy number variation at 11q14.3 is reported to increase susceptibility to nasopharyngeal carcinoma in humans [32]. Loss of 4q34.3 predicts early recurrence of lung adenocarcinoma after adjuvant chemotherapy [33]. Figure 2B reflects the entire genome landscape of ovarian cancer cell line mutations. In this ovarian cancer cell analysis, it was confirmed that there was copy number variation in the chromosome 6q21-qter region. At the same time, there is also a high frequency of copy number variation and loss of heterozygosity (LOH) at the gross histological tumor level in ovarian cancer patients [20, 34, 35]. Considering that the copy number variation of the 6q21-qter region did not show strong significance in this cell result, only the overall genetic structure effect analysis, the 6q21-qter is the chromosome-sensitive region of ovarian cancer, which we focused on. Therefore, we collected 442 genes with copy number variation in the 6q21-qter region for subsequent analysis.

Data analysis based on the Cancer Genome Atlas (TCGA) revealed that somatic changes in ten typical pathways play an important role in tumorigenesis and development [36]. Including Notch, RTK-RAS, Wnt, Hippo, Myc, PI3K, TP53, TGFβ and Cell Cycle pathway. The two ovarian cancer cell lines had the highest frequency of gene mutation in Notch and RTK-RAS pathway, and the lowest frequency of overall change in Cell Cycle signal pathway (Fig. 2C).

### Functional annotation and survival analysis of genes with copy number variations in the 6q21-qter region

**Fig. 3:**
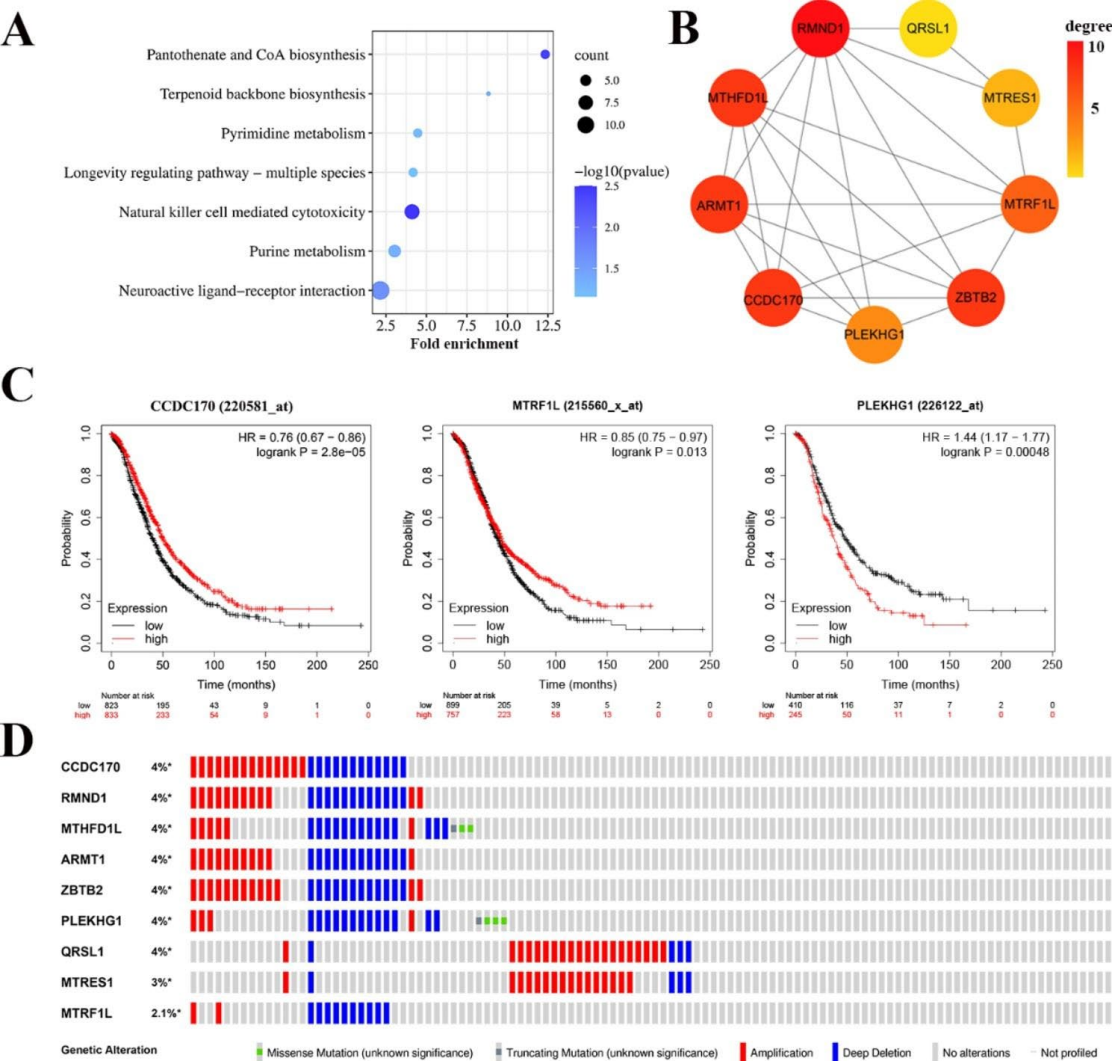
Copy number variation gene analysis of 6q21-qter region. (**A**) KEGG pathway enrichment analysis of 442 copy number variation genes. (**B**) PPI network of 9 hub genes identified in copy number variation genes. (**C**) Kaplan Meier survival analysis of hub genes CCDC170, MTRF1L and PLEKHG1. The statistical logrank P values of CCDC170, MTRF1 L and PLEKHG1 were 0.000028,0.013 and 0.00048, respectively. (**D**) Nine hub gene mutations in three different ovarian cancer cohorts in the cBioPortal database. Each column represents a tumor sample, and each row represents a hub gene

The DAVID online analysis tool was used to biologically annotate 442 genes with copy number variation in the 6q21-qter region, and GO and KEGG enrichment were obtained. BP was mainly enriched in natural killer cell-mediated cytotoxicity; CC was mainly enriched in integral component of membrane; and MF was mainly enriched in protein binding function (Additional file 4: Fig S4). The copy number variation analysis in the 6q21-qter region is based on ovarian epithelial cell lines but the enriched pathway shows multiple immune cell pathway including natural killer cell-mediated cytotoxicity and neuroactive ligand‒receptor interaction (Fig. 3A). We speculate that genetic variation may cause changes in the tumor microenvironment, resulting in immunosuppressive effects of the natural killer cell-mediated cytotoxicity pathway, leading to the carcinogenesis of target cells. The neuroactive ligand-receptor interaction pathway, is currently receiving increasing attention for its role in the immune system. It has been confirmed that this pathway can affect the proliferation, differentiation, apoptosis and angiogenesis of various immunocompetent cells, thus playing a role in malignant tumors, especially ovarian cancer. The GO and KEGG enrichment results showed that copy number variation genes were related to tumor apoptosis, progression and metastasis.

After obtaining the protein‒protein interaction (PPI) data of copy number variation genes using the STRING database, 9 hub genes were screened using the MCODE plug-in in Cytoscape software (Fig. 3B). To explore the association between these hub genes and clinical parameters, Kaplan-Meier Plotter analysis was performed. The differential expression of CCDC170, MTRF1L, ZBTB2, ARMT1, PLEKHG1 and QRSL1 genes affected the overall survival (OS) of ovarian cancer patients (Fig. 3C, Additional file 4: Fig S4). The prognosis of ovarian cancer patients was significantly improved when the CCDC170 gene was highly expressed. At the same time, the expression of CCDC170 in ovarian cancer tissues was significantly lower than that in normal tissues (Additional file 4: Fig S4). Therefore, this is one of the genes we focused on. In addition, we used three research cohorts in the cBioPortal database to observe the mutations of these hub genes. A total of 11% of the 781 sequenced cases showed mutations in at least one of the nine central genes investigated. Figure 3D shows the change frequency of each of the 9 hub genes.

### Functional annotation and survival prognosis analysis of shared mutant genes

**Fig. 4:**
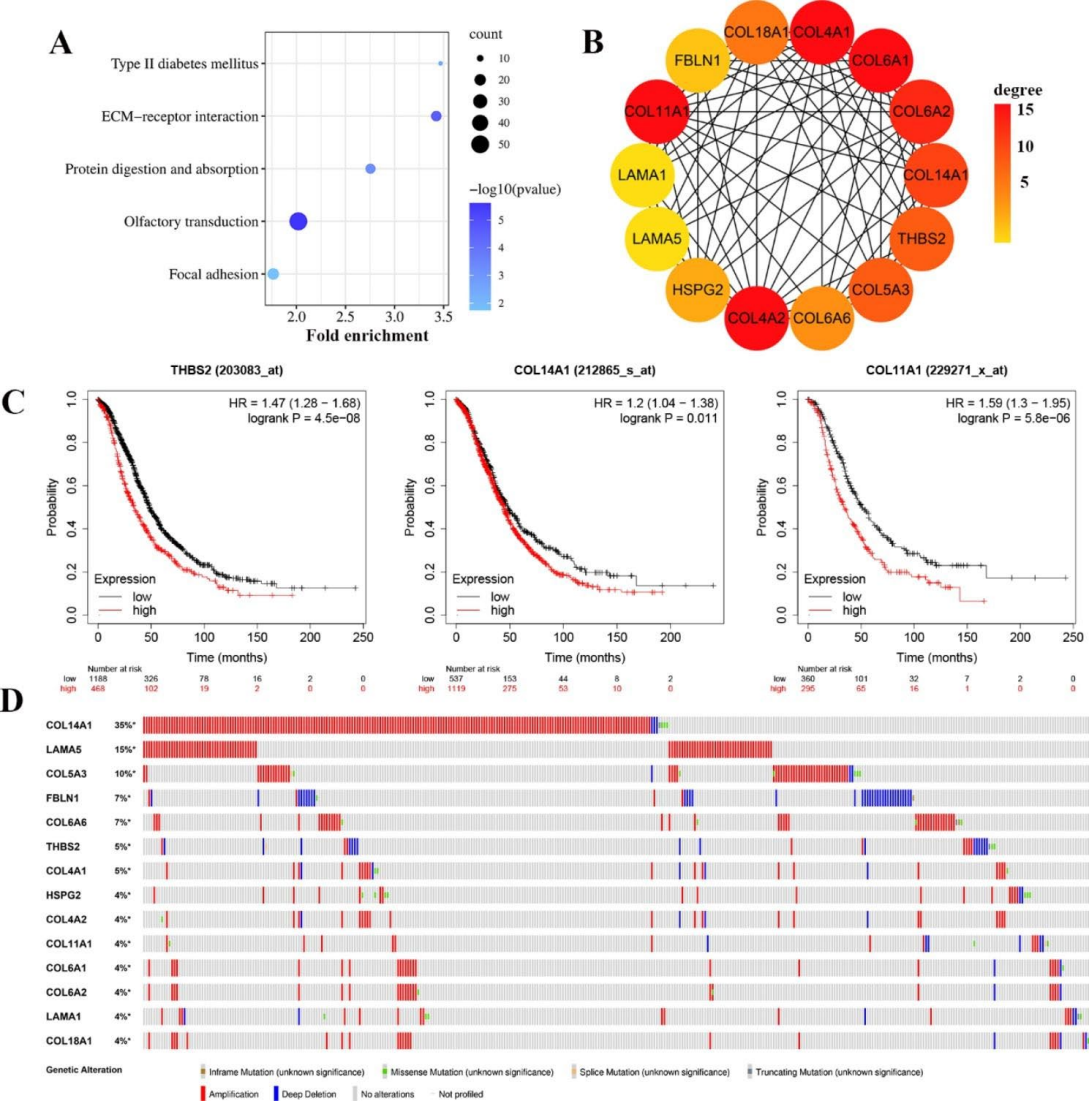
Shared mutation gene analysis. (**A**) KEGG pathway enrichment analysis of 2547 shared mutant genes. (**B**) PPI network of 14 hub genes identified in shared mutant gene. (**C**) Kaplan Meier survival analysis of hub genes THBS2, COL14A1 and COL11A1. (**D**) Fourteen hub gene mutations in three different ovarian cancer cohorts in the cBioPortal database. Each column represents a tumor sample, and each row represents a hub gene

We further explored the common characteristics of mutant genes between mucinous and clear cell ovarian cancer. We identified a total of 2547 shared mutant genes in both cell lines. KEGG and GO analyses of these 2547 genes were performed using the DAVID online tool. In GO analysis, BP was mainly enriched in cell adhesion; CC was mainly enriched in extracellular matrix and cytoskeleton; MF was mainly enriched in calcium ion binding and extracellular matrix structural components (Additional file 5: Fig S5). In addition, in the KEGG signaling pathway analysis, the shared mutant genes were mainly enriched in ECM-receptor interactions and focal adhesion (Fig. 4A). The above results indicate that the shared mutant genes are mainly enriched in pathways closely related to tumor metastasis.

Additionally, 14 hub genes were screened by Cytoscape software (Fig. 4B). Most of these genes are members of the collagen family and are closely related to tumor infiltration. Genes such as COL14A1, COL6A1, THBS2, COL4A2 and COL11A1 are involved in tumor migration and invasion. Survival analysis revealed that the differential expression of 10 hub genes affected the prognosis of ovarian cancer patients (Fig. 4C, Additional file 5: Fig S5). Among them, the THBS2 and COL14A1 genes had the most significant effect on the overall survival of ovarian cancer patients. In addition, in the cBioPortal database, the mutation rate of the COL14A1 gene was as high as 35%, and the mutation rate of other hub genes was not less than 4% (Fig. 4D). Therefore, THBS2 and COL14A1 are also our key molecular targets. A total of 64% of the 781 sequenced cases showed at least one mutation in the 14 hub genes queried. Figure 4D shows the frequency of alterations in each of the 14 hub genes.

### Mutation sites and expression analysis of CCDC170, COL14A1 and THBS2 genes on bioinformatics

**Fig. 5:**
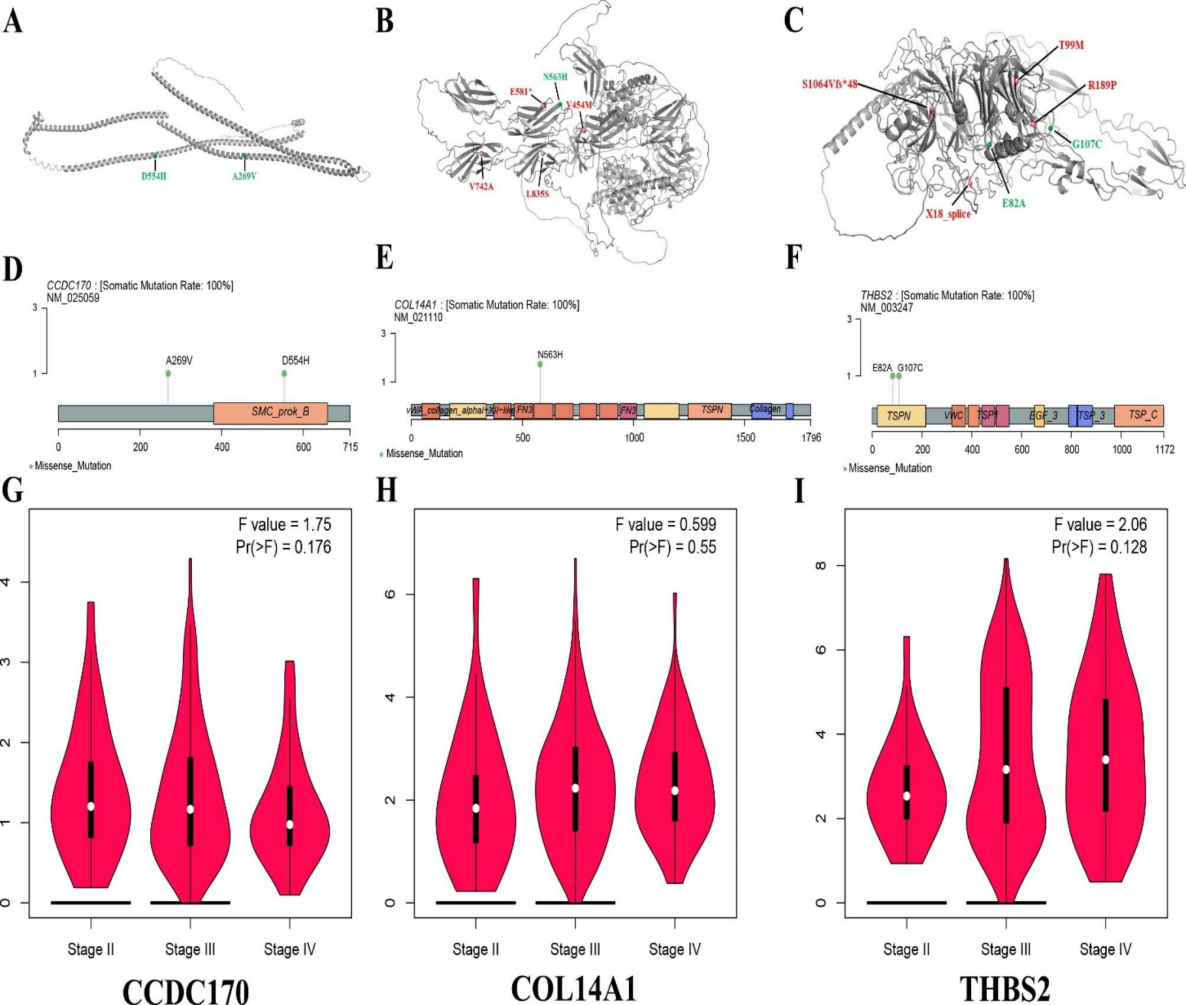
CCDC170, COL14A1 and THBS2 mutation distribution and tumor stage differential expression. (**A**-**C**) AlphaFold structure prediction model based on CCDC170 (**A**), COL14A1 (**B**) and THBS2 (**C**) maps the mutated residues in ovarian cancer cell lines. All amino acid numbers are based on protein sequences. The mutant residues of ovarian cancer cell lines showed green spheres, and the mutant residues of cBioportal cohort showed red spheres. (**D**-**F**) The distribution of CCDC170 (**D**), COL14A1 (**E**) and THBS2 (**F**) somatic mutations identified in two ovarian cancer cells. (**G**-**I**) Differential expression of CCDC170 (**G**), COL14A1 (**H**) and THBS2 (**I**) genes in different tumor stages. The method for differential gene expression analysis is one-way ANOVA. The expression data are first log_2_(TPM + 1) transformed for differential analysis

Gene mutations can affect mRNA, protein levels and protein structure. We further explored the similarities and differences between CCDC170, COL14A1 and THBS2 gene point mutations and cBioPortal database point mutations. We show the putative ovarian cancer-associated SNV with protein annotation in Fig. 5A-F. Similar to the point mutations (V454M, V742A, L835S, E581*) in the cBioPortal database, the COL14A1 point mutation (N56H3) is mainly concentrated in the FN3 domain. Frequent point mutations of the THBS2 gene mainly occurred near sequence 1-200 (ovarian cancer cell lines: E82A and G107E; cBioPortal database: R189P, T99M, X18_ splice). In the three ovarian cancer cohorts, only copy number variation of the CCDC170 gene occurred. Our results showed that CCDC170 had missense mutations in A269V and D554H.

In addition, we also used TCGA data to detect the expression of COL14A1, THBS2 and CCDC170 genes in different stages of ovarian cancer patients. We observed that the expression of COL14A1 and THBS2 in advanced patients was significantly higher than that in early- and middle-stage patients (Fig. 5H-I). In contrast, CCDC170 showed low expression in advanced patients (Fig. 5G). These results are consistent with the above Kaplan-Meier survival analysis. This indicates that CCDC170, COL14A1 and THBS2 are expected to become targets for the diagnosis and treatment of ovarian cancer. We will further verify this through experiments.

### Expression of CCDC170, COL14A1 and THBS2 in ovarian cancer cell lines and normal ovarian epithelial cells by WB and IF experiments

**Fig. 6:**
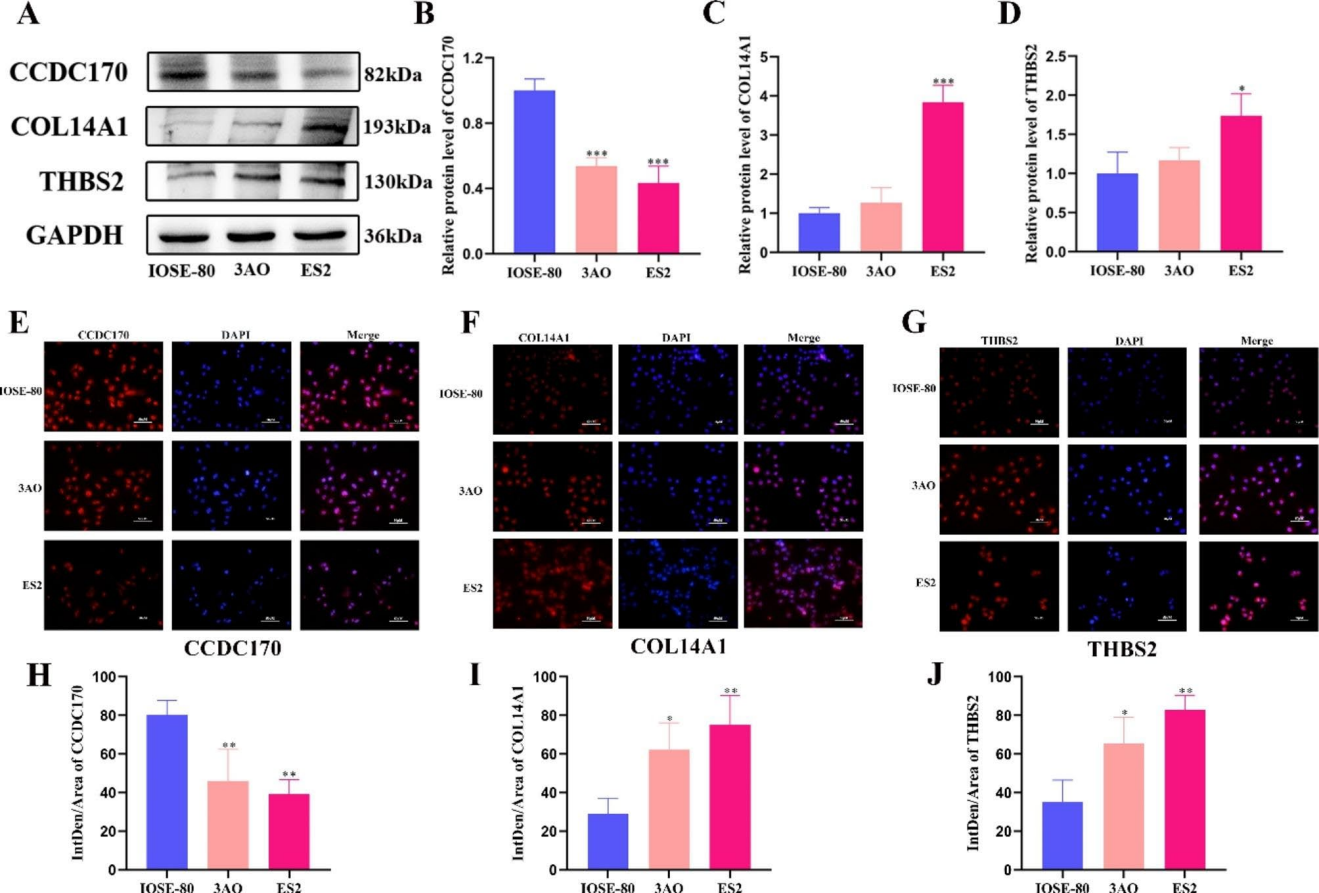
Differential expression of CCDC170, COL14A1 and THBS2 in ovarian cancer cell lines and normal ovarian epithelial cells. (**A**) Western blotting was used to detect the expression of CCDC170, COL14A1 and THBS2 in ovarian cancer cell lines and normal ovarian epithelial cells. In order to improve the clarity and conciseness of the presentation, we cropped the blots. The original, untreated blots images can be found in Additional file 6. (**B**-**D**) Statistical analysis of Western blotting results of CCDC170, COL14A1 and THBS2 proteins. (**E**-**G**) The expression of CCDC170, COL14A1 and THBS2 in ovarian cancer cell lines and normal ovarian epithelial cells was detected by immunofluorescence assay. Original magnification: 40×. (**H**-**J**) Statistical analysis of mean fluorescence density of CCDC170, COL14A1 and THBS2 proteins. The gray values of protein bands and the fluorescence intensity of immunofluorescence were analyzed using Image J software (National Institutes of Health, Bethesda, MD, USA).

We verified the expression of CCDC170, THBS2 and COL14A1 in ovarian cancer cells by Western blotting (WB) and immunofluorescence assay (IF). As shown in Fig. 6A-D, the expression of CCDC170 in ovarian cancer cell lines 3AO and ES2 was significantly lower than that in IOSE-80 (P < 0.001, P < 0.001). The expression of COL14A1 and THBS2 in ES2 cells was significantly higher than that in IOSE-80 cells (P < 0.001, P < 0.05) and increased in 3AO cells, but the results were not significant (P > 0.05). At the same time, the results of the cell immunofluorescence assay were consistent with those of Western blotting. As shown in Fig. 6E-J, the fluorescence intensity of CCD170 in 3AO and ES2 cells was significantly lower than that in IOSE-80 cells (P < 0.01, P < 0.01). In addition, the expression of COL14A1 and THBS2 was significantly increased in ovarian cancer cell lines compared to normal ovarian cells (P < 0.05, P < 0.01), but the differential expression in CCDC170 was the opposite.

## Discussion

Mucinous and clear cell ovarian cancer is a rare subtype of epithelial ovarian cancer with poor prognosis and chemoresistance at advanced stages [37]. Recent studies have found frequent mutations, such as ARID1A and PIK3CA, in mucinous and clear cell ovarian cancer [38, 39]. However, these scientific findings have not yet been effectively translated into prevention and individualized treatment, which remains a major challenge for advanced diagnosis, chemotherapy resistance and the low survival rate of ovarian cancer patients. Ovarian cancer and other tumors, such as liver cancer, breast cancer and cervical cancer, often have copy number deletions or amplifications on the long arm of chromosome 6 [40, 41]. Other studies have also shown that ovarian cancer has unstable aberrations in regions such as chromosome 8, which needs further research to be confirmed. This provides a new orientation and direction for a better understanding of the pathogenic mutations of ovarian cancer and finding markers for diagnosis, treatment and prognosis.

To this end, we performed WES on mucinous and clear cell ovarian cancer cell lines to reveal their mutant landscape and screen molecular markers. A total of 4529 and 4318 variants were detected in 3AO and ES2 cells, and C > T and T > C were two common substitutions (Fig. 1A-C). Through identification, MUC12, FLG and MUC16 genes were found to be highly mutated (Fig. 1F). MUC12 and MUC16 are members of the mucin family. Mucin mutations are frequently observed in malignant cancers and are involved in cancer progression by transducing intracellular signaling [42, 43]. MUC12, for example, is highly mutated and carcinogenic in colorectal cancer and clear cell renal cell carcinoma and promotes tumor invasion [44]. MUC16 (also known as CA125) is overexpressed in a variety of cancers and plays an important role in tumorigenesis and acquired therapeutic resistance [45]. It is mainly used as a marker for the early diagnosis of ovarian cancer [5]. FLG mutations are strongly associated with cervical cancer and cutaneous melanoma and can serve as a biomarker for prognosis and treatment [46]. Ovarian cancer cell lines showed cumulative mutations in the Notch, RTK-RAS, Wnt and TGF-β pathways in ten known carcinogenic signaling pathways (Fig. 2C). MUC12 and MUC16 are primarily involved in the TGF-β and Wnt signaling pathways that promote tumorigenesis and metastasis [31, 47]. Therefore, our genomic variation results are consistent with the literature, indicating that ovarian cancer cell line bioinformatics analysis is accurate and credible. The tumor specificity of MUC12 and FLG is not strong, and the early diagnosis of MUC16 in ovarian cancer is not satisfactory. In particular, molecular markers for the specific diagnosis of mucinous and clear cell ovarian cancer tissue subtypes are still lacking and need to be explored. Therefore, this study carried out the following in-depth hub gene screening work.

We screened 14 hub genes in the 2547 shared mutant gene set (Fig. 4B). Among them, 9 genes belong to the collagen family. Kaplan-Meier survival curve analysis showed that ovarian cancer patients with high expression of collagen family genes had poor prognosis (Fig. 4C, Additional file 5: Fig S5). In particular, the mutation rate of the COL14A1 gene was as high as 35% in 781 clinical samples from three study cohorts (Fig. 4D). The expression of COL14A1 in advanced ovarian cancer patients is significantly higher than that in early and middle ovarian cancer patients, which significantly affects the prognosis of patients. LIU Z and RøMER et al. reported that collagen is upregulated in a variety of cancers, including ovarian cancer, breast cancer, thyroid cancer, pancreatic cancer, non-small cell lung cancer and bladder transitional cell carcinoma [48, 49]. Collagen is the main component of the extracellular matrix (ECM), which can enhance ECM stiffness, promote angiogenesis and guide infiltration and play an important role in tumor metastasis [50, 51]. In addition, the results of our Kaplan-Meier analysis showed that high expression of THBS2 significantly affected overall survival in patients with ovarian cancer (p < 0.001) (Fig. 4C). According to the literature, THBS2 is frequently mutated in a variety of cancers, and its high expression promotes tumor growth and distant metastasis [52, 53]. These data suggest that the shared mutant genes of the two cell lines may be related to the invasion and dissemination of ovarian cancer.

Copy number analysis showed that 3AO and ES2 cells were mainly amplified at 4p16.1 and 11q14.3, and copy number loss occurred at 4q34.3 and 18p11.21 (Fig. 2A). Copy number amplification and deletion were also confirmed in chromosome 6q21-qter (See orange data in Fig. 2A). Our previous work also confirmed that there is a loss of heterozygosity in the 6q21-qter region of ovarian cancer, which is the chromosome-sensitive region of the tumor. According to the literature, 6q21-qter instability (such as chromosome deletion) significantly affects the survival prognosis of patients with ovarian cancer [35, 54]. Considering that the results of ovarian cancer cell-level research and gross histological tumor levels were consistent and supported each other, we further confirmed that there are frequent 6q21-qter copy number amplifications and deletions in the ovarian cancer genome, so we mainly studied this region. We screened 9 hub genes out of 442 genes with copy number variations in the 6q21-qter region (Fig. 3B). CCDC170 was the most significantly associated gene with ovarian cancer prognosis (p < 0.001) (Fig. 3C). CCDC170 is a coiled-coil domain-containing protein that affects apoptosis of breast cancer cells through the IRE1 pathway, and a few studies have reported that ESR1-CCDC170 fusion is a carcinogenic fusion driver for breast cancer [55, 56]. We identified two novel missense mutations (A269V, D554H) in mucinous and clear cell ovarian cancer (Fig. 6A). It was first reported that CCDC170 copy number variation can reduce its expression, thereby affecting the overall survival of patients with ovarian cancer, and it is a pathogenic mutation of ovarian cancer. At present, agonists such as luteinizing hormone releasing hormone, gonadotropin-releasing hormone and natural estrogen receptor β have shown beneficial effects on the treatment of ovarian cancer [57–59]. As a newly discovered downregulated molecular target of ovarian cancer, CCDC170 can provide a new direction for the development of agonists to reverse the treatment of ovarian cancer.

Based on the above analysis, we selected three molecular markers (CCDC170, COL14A1, THBS2) that are expected to be used in clinical ovarian cancer and verified them by Western blotting and immunofluorescence experiments. In our Western blotting results, the expression of CCDC170 in ovarian cancer cell lines was significantly lower than that in normal ovarian cells (Fig. 6A-D). Moreover, the same experimental results appeared in the cell immunofluorescence experiments (Fig. 6E). The results in Fig. 6E showed that CCDC170 was highly expressed in the nucleus and cytoplasm of IOSE-80 and 3AO cells. Compared with IOSE80 cells, the expression of CCDC170 in the cytoplasm of 3AO cells was relatively low. The same is true for ES2 cells. The possible reason is that the expression of CCDC170 in the cytoplasm of tumor cells is inhibited, leading to tumor progression. These experimental results are consistent with our bioinformatics analysis. Similarly, although THBS2 and COL14A1 also showed differential expression in ovarian cancer cell lines and normal ovarian cells (Fig. 6A, F-G), the results were not as significant as those for CCDC170.

## Conclusions

In conclusion, through WES, bioinformatics analysis and cell experiments, a new pathogenic mechanism of ovarian cancer was revealed. The expression of CCD170 in normal tissues was higher than that in tumor tissues (Fig. S4C). At the same time, the expression of CCDC170 in patients with advanced ovarian cancer was lower than that in patients with early ovarian cancer (Fig. 5G). The survival curve of Fig. 3C also shows that ovarian cancer patients with high expression of CCDC170 have better prognosis. CCDC170 can be used as a new promising molecular target for ovarian cancer patients, which is related to the good prognosis of the protein.

## Electronic supplementary material

Below is the link to the electronic supplementary material.

Supplementary Material 1

Supplementary Material 2

Supplementary Material 3

Supplementary Material 4

Supplementary Material 5

Supplementary Material 6

## Data Availability

The datasets generated and/or analysed during the current study are available in the GenBank repository, https://www.ncbi.nlm.nih.gov/sra/PRJNA929457, accession: PRJNA929457. The original data of the study are available from the corresponding authors upon reasonable request.

## Abbreviations

OS: overall survival
SNP: single nucleotide polymorphism
CNV: copy number variation
WES: whole exome sequencing
GO: gene ontology
KEGG: kyoto encyclopedia of genes and genomes
OC: ovarian cancer
CCDC170: coiled-coil domain-containing 170
COL14A1: collagen type XIV alpha 1 chain
THBS2: thrombospondin 2
PPI: protein-protein interactions
SOC: serous ovarian carcinoma
MOC: mucinous ovarian carcinoma
OEC: ovarian endometrioid carcinoma
OCCC: Ovarian clear cell carcinoma
PVDF: polyvinylidene fluoride
TCGA: the cancer genome atlas
GEO: gene expression omnibus
DAPI: 4',6-diamidino-2-phenylindole
ECM: extracellular matrix
GATK: genome analysis toolkit
