# Supplementary material for: Whole-exome mutational landscape and molecular marker study in mucinous and clear cell ovarian cancer cell lines 3AO and ES2
Source: BMC Cancer. 2023 Apr 6;23:321. doi: 10.1186/s12885-023-10791-9 (PMC10080944; doi:10.1186/s12885-023-10791-9)
Supplement: Supplementary file 1 — Supplementary Material 1 [file 12885_2023_10791_MOESM1_ESM.pdf]

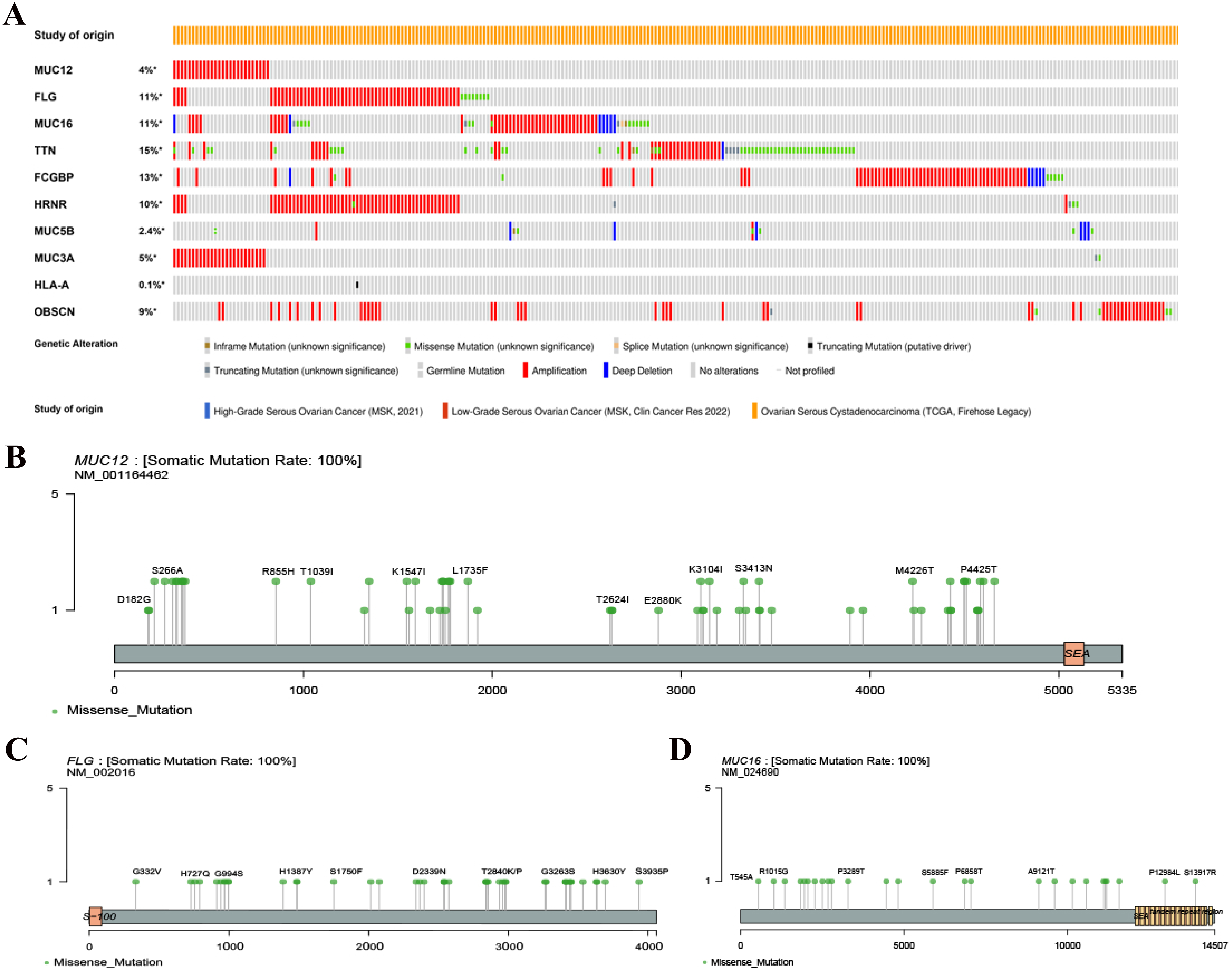
A
Study of origin
MUC12 4%*
FLG 11%*
MUC16 11%*
TTN 15%*
FCGBP 13%*
HRNR 10%*
MUC5B 2.4%*
MUC3A 5%*
HLA-A 0.1%*
OBSCN 9%*
Genetic Alteration
Inframe Mutation (unknown significance)
Missense Mutation (unknown significance)
Splice Mutation (unknown significance)
Truncating Mutation (putative driver)
Truncating Mutation (unknown significance)
Germline Mutation
Amplification
Deep Deletion
No alterations
Not profiled
Study of origin
High-Grade Serous Ovarian Cancer (MSK, 2021)
Low-Grade Serous Ovarian Cancer (MSK, Clin Cancer Res 2022)
Ovarian Serous Cystadenocarcinoma (TCGA, Firehose Legacy)
B
MUC12 : [Somatic Mutation Rate: 100%]
NM_001164462
5
1
D182G
S266A
R855H
T1039I
K1547I
L1735F
T2624I
E2880K
K3104I
S3413N
M4226T
P4425T
SEA
0
1000
2000
3000
4000
5000
5335
Missense_Mutation
C
FLG : [Somatic Mutation Rate: 100%]
NM_002016
5
1
G332V
H727Q
G994S
H1387Y
S1750F
D2339N
T2840K/P
G3263S
H3630Y
S3935P
S-100
0
1000
2000
3000
4000
Missense_Mutation
D
MUC16 : [Somatic Mutation Rate: 100%]
NM_024690
5
1
T545A
R1015G
P3289T
S5885F
P6858T
A9121T
P12984L
S13917R
SEA
Tandem repeat region
0
5000
10000
14507
Missense_Mutation

Figure S1. Point mutation distribution of MUC12, FLG and MUC16 in ovarian cancer cells and Top10 gene mutation in cBioPortal ovarian cancer cohort. (A) Top10 gene mutations in three different ovarian cancer cohorts in the cBioPortal database. Each column represents a tumor sample, and each row represents a hub gene. (B-D) The distribution of MUC12 (B), FLG (C) and MUC16 (D) somatic mutations identified in two ovarian cancer cells.
