# Supplementary material for: Whole-exome mutational landscape and molecular marker study in mucinous and clear cell ovarian cancer cell lines 3AO and ES2
Source: BMC Cancer. 2023 Apr 6;23:321. doi: 10.1186/s12885-023-10791-9 (PMC10080944; doi:10.1186/s12885-023-10791-9)
Supplement: Supplementary file 2 — Supplementary Material 2 [file 12885_2023_10791_MOESM2_ESM.pdf]

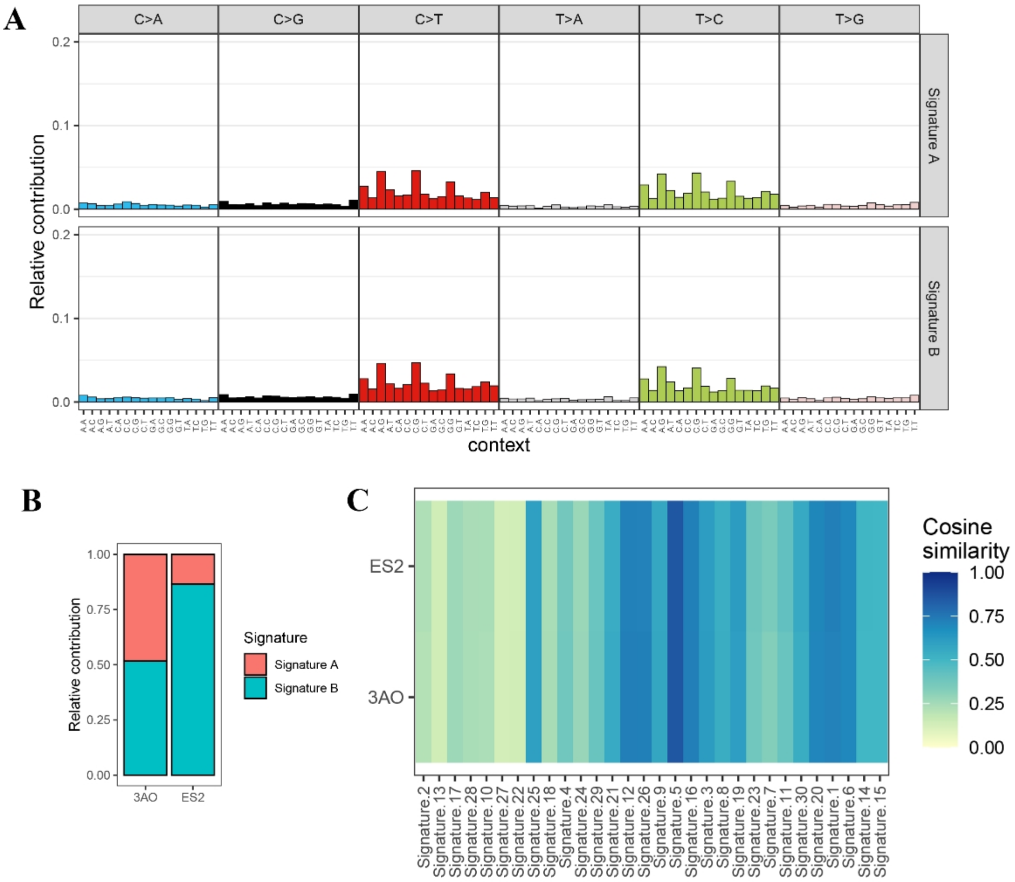

Figure S2. Mutational signature comparison of 3AO and ES2 cells. (A) Mutational signatures A and B. The mutation spectrum of the two cell lines was decomposed into different mutation signatures by NMF method. Each color refers to one of six base substitutions. (B) Mutational signature exposure ratio for 3AO and ES2 cells. (C) The cosine similarity with 30 known mutation signatures in the COSMIC database. Dark blue represents high cosine similarity, and light yellow represents low cosine similarity.
