# Supplementary material for: Whole-exome mutational landscape and molecular marker study in mucinous and clear cell ovarian cancer cell lines 3AO and ES2
Source: BMC Cancer. 2023 Apr 6;23:321. doi: 10.1186/s12885-023-10791-9 (PMC10080944; doi:10.1186/s12885-023-10791-9)
Supplement: Supplementary file 3 — Supplementary Material 3 [file 12885_2023_10791_MOESM3_ESM.pdf]

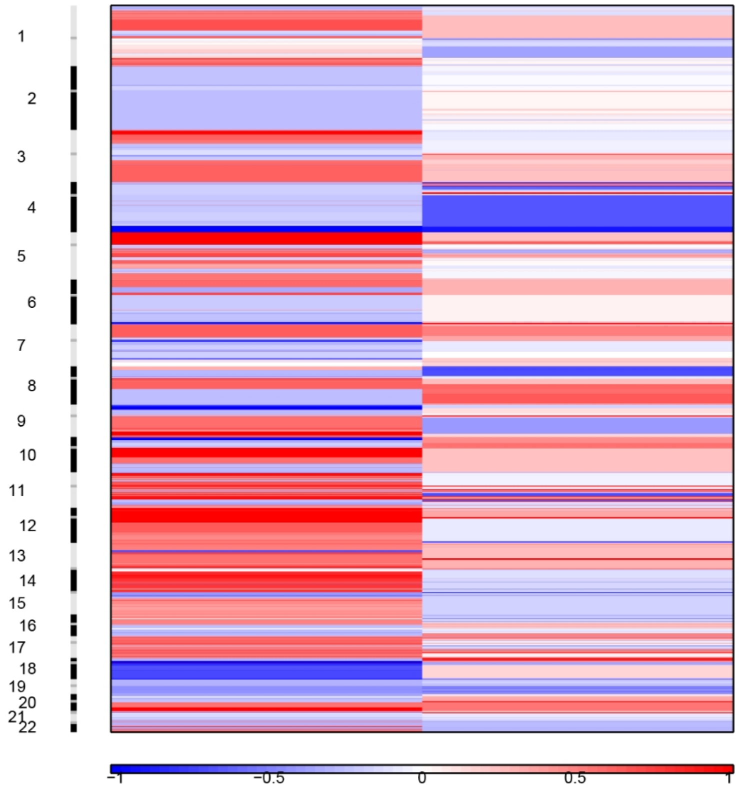

Figure S3. Copy number variation landscape of 3AO and ES2 cells. Red represents the amplification of genome copy number, and blue represents the deletion of genome copy number.
