# Supplementary material for: Whole-exome mutational landscape and molecular marker study in mucinous and clear cell ovarian cancer cell lines 3AO and ES2
Source: BMC Cancer. 2023 Apr 6;23:321. doi: 10.1186/s12885-023-10791-9 (PMC10080944; doi:10.1186/s12885-023-10791-9)
Supplement: Supplementary file 4 — Supplementary Material 4 [file 12885_2023_10791_MOESM4_ESM.pdf]

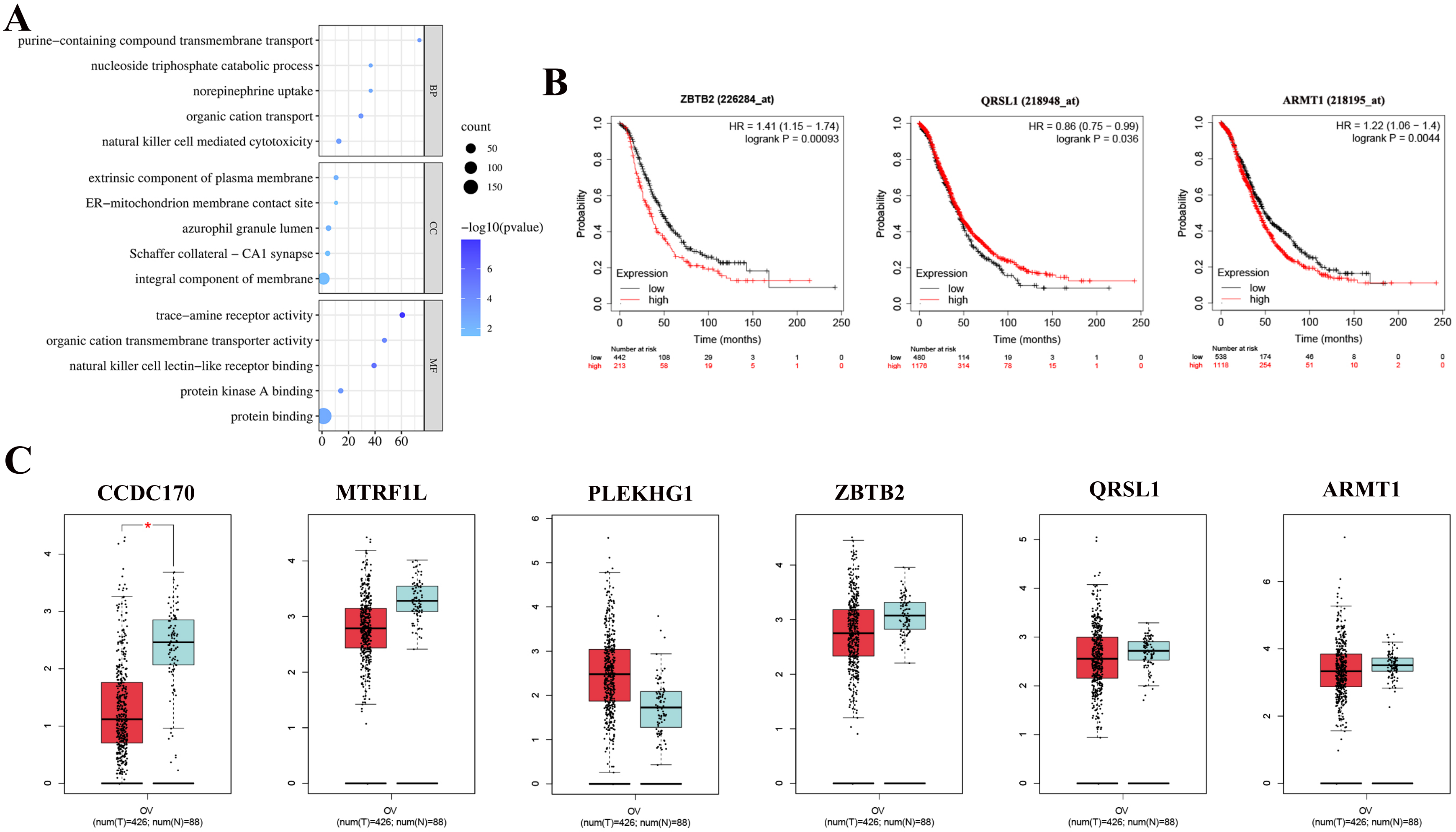
A
purine−containing compound transmembrane transport
nucleoside triphosphate catabolic process
norepinephrine uptake
organic cation transport
natural killer cell mediated cytotoxicity
BP
extrinsic component of plasma membrane
ER−mitochondrion membrane contact site
azurophil granule lumen
Schaffer collateral – CA1 synapse
integral component of membrane
CC
trace−amine receptor activity
organic cation transmembrane transporter activity
natural killer cell lectin−like receptor binding
protein kinase A binding
protein binding
MF
0 20 40 60
count
50
100
150
−log10(pvalue)
6
4
2
B
ZBTB2 (226284_at)
HR = 1.41 (1.15 − 1.74)
logrank P = 0.00093
Probability
Time (months)
Expression
low
high
Number at risk
low 442 108 29 3 1 0
high 213 58 19 5 1 0
QRSL1 (218948_at)
HR = 0.86 (0.75 − 0.99)
logrank P = 0.036
Number at risk
low 480 114 19 3 1 0
high 1176 314 78 15 1 0
ARMT1 (218195_at)
HR = 1.22 (1.06 − 1.4)
logrank P = 0.0044
Number at risk
low 538 174 46 8 0 0
high 1118 254 51 10 2 0
C
CCDC170
MTRF1L
PLEKHG1
ZBTB2
QRSL1
ARMT1
*
OV
(num(T)=426; num(N)=88)

Figure S4. Prognostic and expression analysis of copy number variation hub genes. (A) GO analysis of 442 copy number variation genes. (B) Kaplan Meier survival analysis of hub genes ZBTB2, QRSL1 and ARMT1. (C) The differential expression of CCDC170, MTRF1L, PLEKHG1, ZBTB2, QRSL1 and ARMT1 genes in ovarian cancer tissues and normal tissues.
