# Supplementary material for: Whole-exome mutational landscape and molecular marker study in mucinous and clear cell ovarian cancer cell lines 3AO and ES2
Source: BMC Cancer. 2023 Apr 6;23:321. doi: 10.1186/s12885-023-10791-9 (PMC10080944; doi:10.1186/s12885-023-10791-9)
Supplement: Supplementary file 5 — Supplementary Material 5 [file 12885_2023_10791_MOESM5_ESM.pdf]

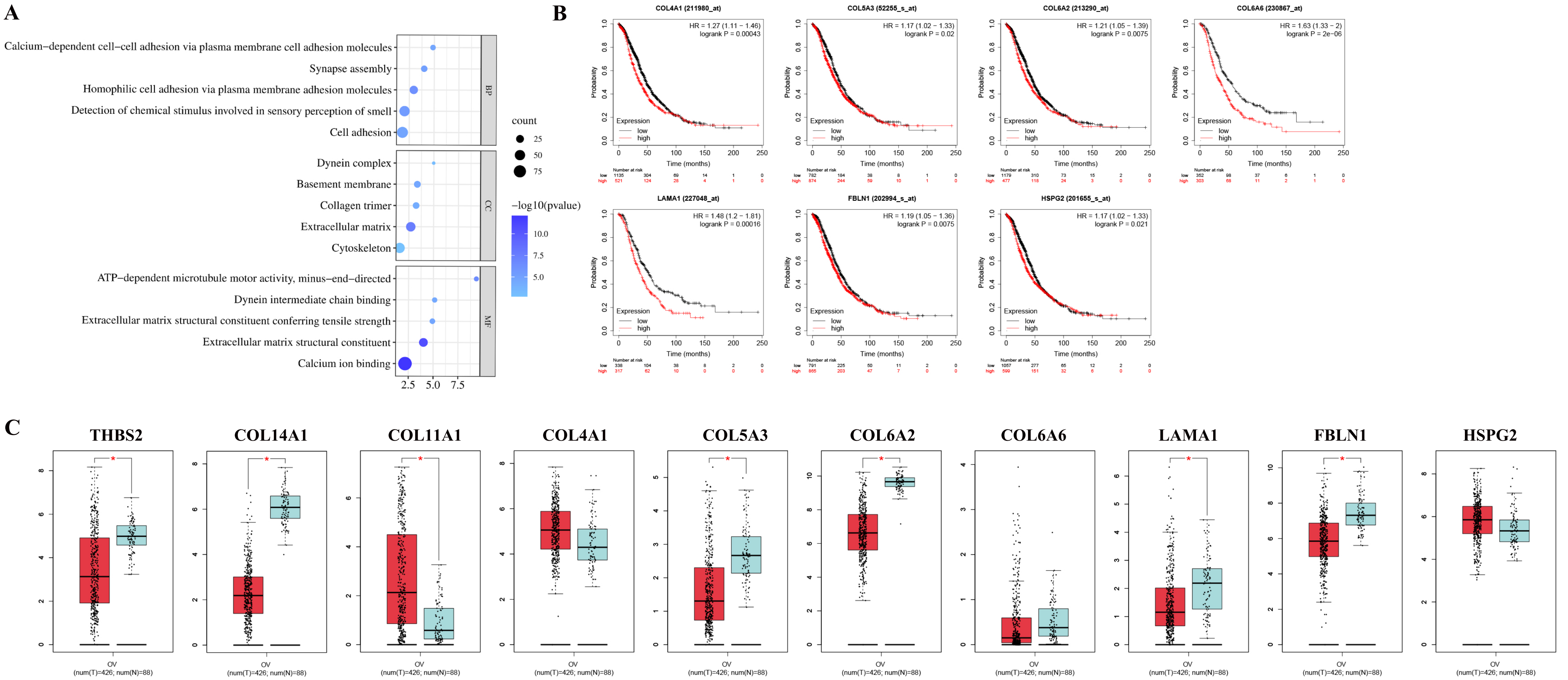
A
Calcium−dependent cell−cell adhesion via plasma membrane cell adhesion molecules
Synapse assembly
Homophilic cell adhesion via plasma membrane adhesion molecules
Detection of chemical stimulus involved in sensory perception of smell
Cell adhesion
BP
Dynein complex
Basement membrane
Collagen trimer
Extracellular matrix
Cytoskeleton
CC
ATP−dependent microtubule motor activity, minus−end−directed
Dynein intermediate chain binding
Extracellular matrix structural constituent conferring tensile strength
Extracellular matrix structural constituent
Calcium ion binding
MF
2.5 5.0 7.5
count
25
50
75
−log10(pvalue)
10.0
7.5
5.0
B
COL4A1 (211980_at)
HR = 1.27 (1.11 − 1.46)
logrank P = 0.00043
COL5A3 (52255_s_at)
HR = 1.17 (1.02 − 1.33)
logrank P = 0.02
COL6A2 (213290_at)
HR = 1.21 (1.05 − 1.39)
logrank P = 0.0075
COL6A6 (230867_at)
HR = 1.63 (1.33 − 2)
logrank P = 2e−06
LAMA1 (227048_at)
HR = 1.48 (1.2 − 1.81)
logrank P = 0.00016
FBLN1 (202994_s_at)
HR = 1.19 (1.05 − 1.36)
logrank P = 0.0075
HSPG2 (201655_s_at)
HR = 1.17 (1.02 − 1.33)
logrank P = 0.021
Probability
Time (months)
Expression
low
high
Number at risk
C
THBS2
COL14A1
COL11A1
COL4A1
COL5A3
COL6A2
COL6A6
LAMA1
FBLN1
HSPG2
OV
(num(T)=426; num(N)=88)

Figure S5. Prognosis and expression analysis of shared mutation hub gene. (A) GO analysis of 2547 shared mutant genes. (B) Kaplan Meier survival analysis of hub genes COL4A1, COL5A3, COL6A2, COL6A6, LAMA1, FBLN1 and HSPG2. (C) The differential expression of THBS2, COL14A1, COL11A1, COL4A1, COL5A3, COL6A2, COL6A6, LAMA1, FBLN1 and HSPG2 genes in ovarian cancer tissues and normal tissues.
