# Supplementary material for: Whole-exome mutational landscape and molecular marker study in mucinous and clear cell ovarian cancer cell lines 3AO and ES2
Source: BMC Cancer. 2023 Apr 6;23:321. doi: 10.1186/s12885-023-10791-9 (PMC10080944; doi:10.1186/s12885-023-10791-9)

Example of original western blot for three repeats

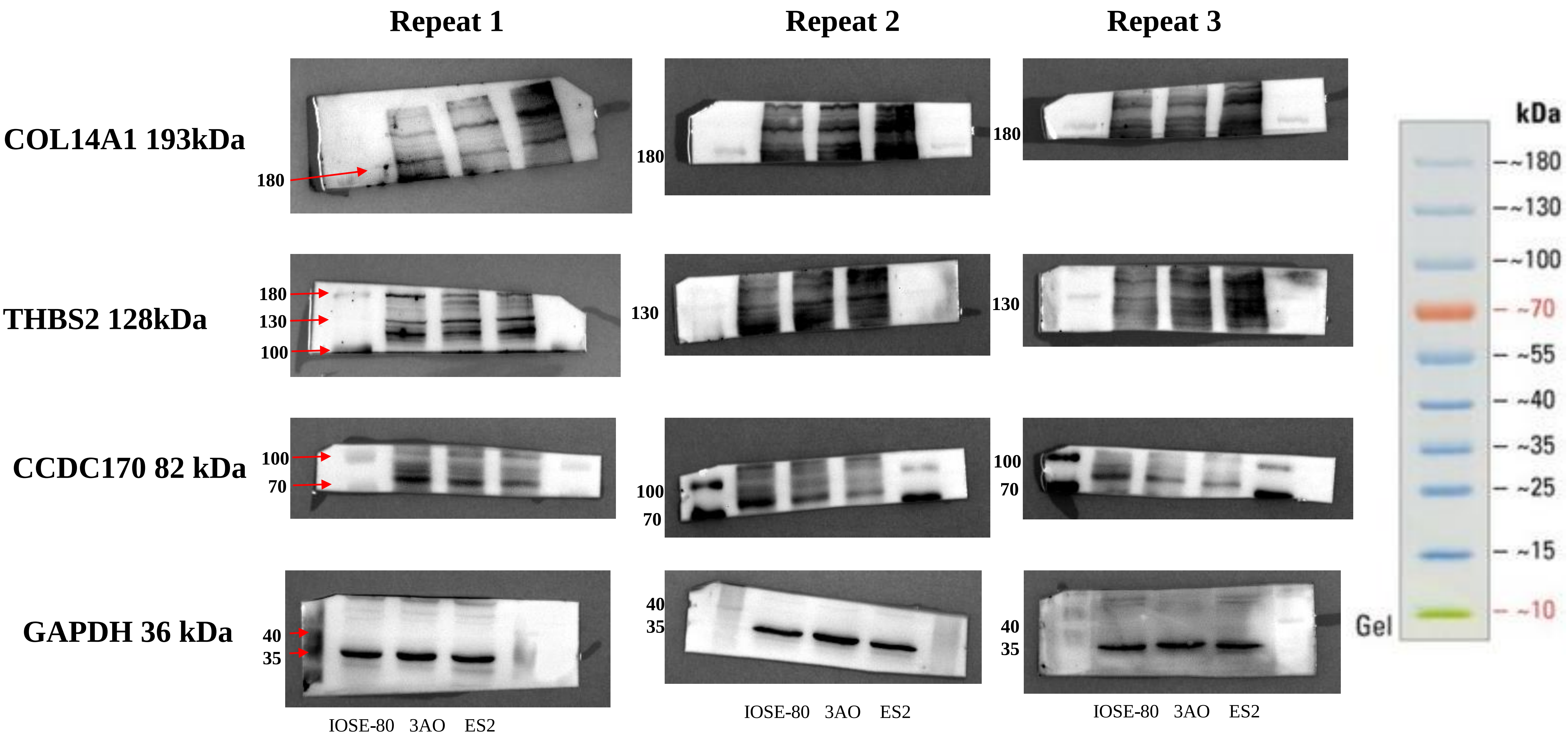

Supplement: Supplementary file 6 — Supplementary Material 6 [file 12885_2023_10791_MOESM6_ESM.pdf]
